# Starch-Water Applications in Burns and Scalds

**Published:** 1866-05

**Authors:** 


*Starch-Water Applications in Burns and Scalds.*—Among the many applications suggested to allay the smarting, to control the troublesome heat and pains of minor burns, or to mitigate the irritability and extreme suffering of severe scalds, partly worn domestics, soaked in *cold* iced starch-water, so applied as to exclude the atmosphere, and continually kept wet by dropping the water on it with a sponge or otherwise, will have a doubly beneficial effect—temporary relief and the keeping of the temperature continuously low, and thereby holding the inflammatory process in abeyance. (Often, if the bowels are watched and made to act freely, there will be but slight inflammation.) When the pain and heat are measurably controlled, which will be in two to four days, the removal of the cloths, and the application of the glycerole of lead, with a camel's hair pencil, or by cloths saturated in it, will result in a speedy cure by resolution, without much vesication, or the formation of ulcers, or secretions of pus. The advantage of starch-water and cloths, over dressings by cotton wadding, oil and lime water, I conceive to be, that it is cooling, while the latter is heating; less bulky; will not, if properly kept wet, adhere to the denuded parts; can be, if required, readily dressed by the attendant, without much trouble or undue pain, because it is always accessible, and should decided suppuration supervene, the fetid odor will not become disagreeable because of the difficulty of removal.—*Medical and Surgical Monthly.*

